# Novel gene encoding a unique luciferase from the fireworm *Odontsyllis undecimdonta*

**DOI:** 10.1038/s41598-018-31086-1

**Published:** 2018-08-24

**Authors:** Yasuo Mitani, Rie Yasuno, Minato Isaka, Nobutaka Mitsuda, Ryo Futahashi, Yoichi Kamagata, Yoshihiro Ohmiya

**Affiliations:** 1 Bioproduction Research Institute, National Institute of Advanced Industrial Science and Technology (AIST), Tsukuba, 305-8566 Japan; 2 Biomedical Research Institute, AIST, Tsukuba, 305-8566 Japan; 3 Graduate School of Science and Technology, Saitama University, Saitama, 338-8570 Japan; 4 DAILAB, Biomedical Research Institute, AIST, Tsukuba, 305-8566 Japan

## Abstract

Luciferases identified or engineered so far emit violet, blue, blue-green, green, yellow, red or near infra-red light. The unique and beautiful bluish-green bioluminescence of fireworms *Odontosyllis* spp. has attracted particular interest, however, their molecular basis is totally unknown partly due to the difficulty of animal collection. Here we report a novel type of luciferase gene from the Japanese fireworm *O. undecimdonta*. The major SDS-PAGE band of the luminous mucus showed luciferase activity. A highly sensitive mass spectrometry analysis in combination with RNA sequencing technique revealed that this band was product of a single gene with no homology to any other sequences in public databases. The recombinant protein of this putative luciferase gene expressed in mammalian cells produced the same unique bluish-green emission peak as the fireworm crude extract, indicating that this novel gene is the genuine fireworm luciferase with an evolutionary different origin from other luciferases previously described. Our findings extend the repertoire of luciferin/luciferase system to previously unavailable wavelength range.

## Introduction

The fascinating luminescence of the fireworms *Odontsyllis* spp., including *O. enopla*, *O. phosporea*, *O. luminosa*, and *O. undecimdonta*, has been described from several places scattering all over the world, Bermuda, the west coast of the North America, Belize, and Japan, and their unique mating behavior with the emission of bluish-green light attracted people’s attention^1–4^. The female comes up to the sea surface and starts to swim in a circle and the male swims directly to the circle center to mate with the female. Most species are known to follow the lunar periodicity and appear at the sea surface a couple of days after the full moon for a several consecutive days^1,2,5–7^. The Japanese species, *O. undecimdonta* is exceptional and comes to the sea surface independent of the lunar cycle in the beginning of October^4^.

Bioluminescence systems have been revealed from a diverse array of animals, as many as more than 10 phyla, using abundant starting materials that were subjected to conventional purification procedures, including column chromatography^8^. Among the phylum Annelida, only two earth worm luciferin have been revealed, and no luciferase or photoprotein have been identified so far^9–11^. In the case of *Odontosyllis* spp., the lunar cycle dependent behavior makes it difficult to obtain a substantial number of animals, which hampers the elucidation of the molecular and chemical aspects of this luminescence system. Shimomura *et al*. estimated that at least 200,000 worms would be needed to obtain fully pure luciferin and luciferase^12^. Using a fourth of this desired amount, they successfully performed a partial purification of the luciferase and obtained highly purified luciferin^12^. Although the characteristics of luciferin, including its absorption spectra and its emission peak at 510 nm, have been revealed^12,13^, its chemical structure and the mechanism of the luminescence reaction including the gene encoding luciferase have not yet been elucidated.

In this study, by combining two technologies, de novo peptide sequencing using highly sensitive mass spectrometry (MS) and RNA sequencing (RNA-seq) using next generation sequencer, we identified a candidate gene, *GoLuc*, for luciferase in Japanese fireworm *O. undecimdonta*. This gene exhibited no homology to any sequence registered in public databases. We successfully confirmed its luminescence activity using mammalian and plant expression systems. The emission spectrum of the recombinant protein was almost identical to that of a crude protein extract of *O. undecimdonta*. Because the emission peak (~510 nm) of fireworm luciferase is different from that of previously characterized luciferase of other animals, our finding will extend the potentially utility of luciferin/luciferase system.

## Results

### Collection of the fireworm in Toyama Bay and the luciferin-luciferase (LL) reaction

**Figure 1:**
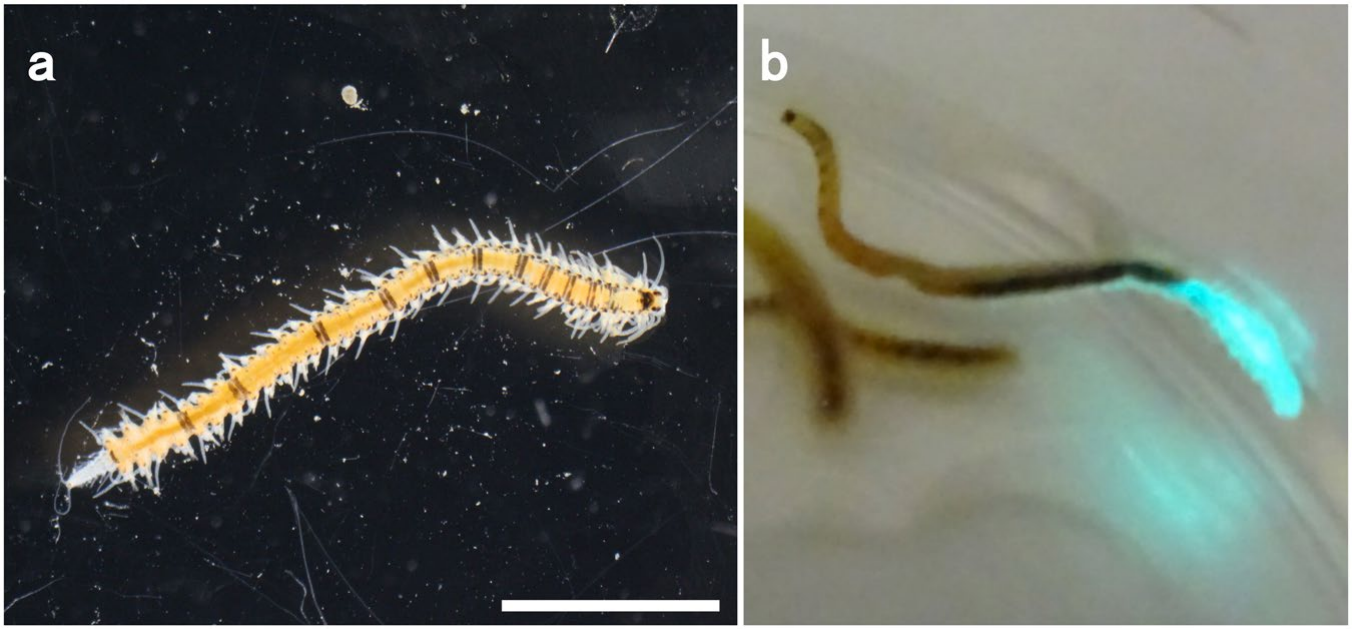
Japanese fireworm *Odontosyllis undecimdonta* obtained from Toyama Bay. (**a**) Brightfield image. (**b**) The worm secreting luminous mucus. Scale bar = 2 mm.

We collected fireworms in the autumn after sunset around Toyama Bay, Japan. The species in Toyama has been regarded to be *O*. *undecimdonta*, which were easily identified due to their light emission and the black stripes on the body (Fig. 1a)^14^. This species appears only at the beginning of October in Toyama Bay. We did not observe natural mating behavior; however, the animals came to the sea surface when a flashlight beam was directed at the surface of the water. The fireworms came to the sea surface 30 to 40 min after sunset and suddenly disappeared approximately 30 min after the appearance of the first worm, like subtropical species. Unlike subtropical species, this species appeared to surface independent of the lunar cycle. We were able to maintain the animals for at least a couple of weeks in aerated sea water in our lab. In the lab, the animals emitted bright bluish-green light when stimulated by a needle tip (Fig. 1b).

**Figure 2:**
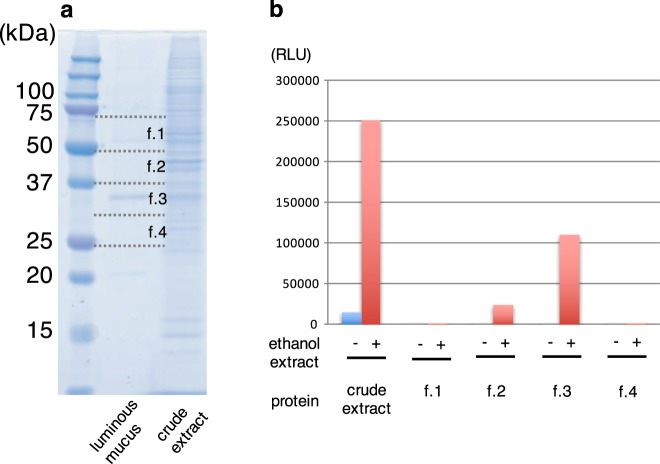
SDS-PAGE analysis and luminescence activity of fireworm. (**a**) Luminous mucus and crude extract were analyzed on a gel. Four gel fractions (f.1–f.4) were used for the luminescence activity. (**b**) Luminescence activity in crude extract and four gel fractions. Ethanol extract of the worms was used as a substrate for the activity assay.

The *Odontosyllis* LL reaction was reported by Harvey using two different species, *O. phosphorea* and *O. enopla* from British Columbia and Bermuda, respectively^15,16^. According to the method of these papers, we prepared ethanol and buffer extracts of the frozen *O*. *undecimdonta*, and confirmed light emission by mixing these two extracts (Fig. 2b, crude extract). Each crude extract containing luciferin or luciferase, respectively, was very stable and emitted light when mixed after at least three months of storage in a freezer. We next investigated the possible cross-reactivity using different synthetic luciferins, including *Cypridina* luciferin, coelenterazine, and furinazine. Additionally, the ethanol extract was subjected to a cross-reactivity assay using recombinant *Cypridina* luciferase^17^. For any combination examined, we did not observe light emission.

### Separation of putative luciferase on a gel

We performed SDS-PAGE analysis for the crude extract containing luciferase activity, because we expected high expression level of luciferase. The resulting gel showed many bands with different intensities, and it was not possible to determine the strongest one (Fig. 2a, right). Next, we carefully stimulated a single animal (Fig. 1b), and collected luminous mucus using a pipet tip. This luciferase-containing solution exhibited a major band at approximately 35 kDa on SDS-PAGE gel with high reproducibility (Fig. 2a, middle). To confirm whether this major band was derived from luciferase or not, we cut the gel into four different pieces before staining the gel and measured the luciferase activities (Fig. 2a,b). Strong luciferase activity was detected around 35 kDa protein, suggesting that this protein is a fireworm luciferase.

### Identification of putative luciferase gene

To identify the putative luciferase protein, the 35 kDa protein was subjected to highly sensitive MS, which showed several strong peptide peaks derived from this protein (Fig. S1). The peaks, m/z = 970.5 and 1075.5 were subjected to MS/MS analysis to obtain amino acid sequence information. The MS/MS analysis predicted two possible amino acid sequences for these two peaks: NVVPLWSR and WEDWVNAR, respectively (Figs S2 and S3). In the case of the latter peak, some variations in the amino acid sequences were also predicted.

**Figure 3:**
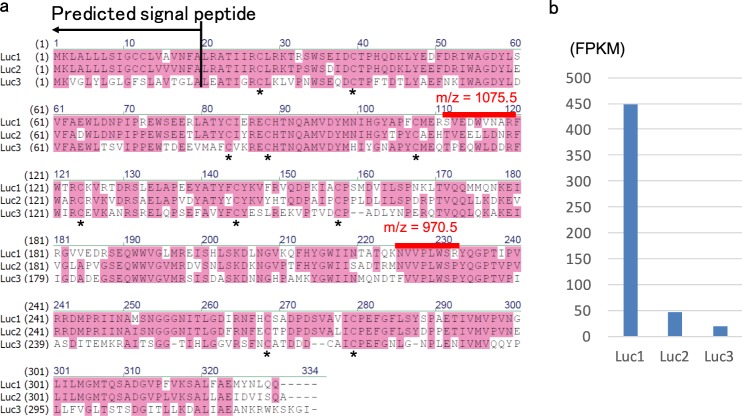
Amino acid sequences and expression analysis of luciferase candidate genes. (**a**) An alignment of a luciferase gene obtained from *Odontosyllis undecimdonta* (Luc1) and its possible paralogs (Luc2, Luc3). Asterisks indicate conserved cysteine residues within putative secreted regions. Signal peptide sequence and peptide sequences identified by MS analysis are also shown. (**b**) Expression levels of three luciferase or luciferase-like genes in whole body based on RNAseq.

In parallel with the MS analysis, RNA-seq was performed by using whole animals. RNA-seq database made it possible to identify the genes containing the amino acid sequences predicted by MS. We found that only a single protein sequence matched with the both of two predicted amino acid sequences (Fig. 3a). One of the predicted amino acid sequences was WEDWVNAR, but that in the protein sequence was SVEDWVNAR. This difference was probably due to the similarity of molecular weight between W and S plus V. We also confirmed its nucleotide sequence by RT-PCR (accession number: LC333365). Genome PCR clarified that this gene consists of 8 exons in about 5 kb genomic region (Fig. S4, accession number: LC333366). This protein (hereafter called Luc1) contained 329 amino acids with putative signal peptide sequence at the N-terminus (Fig. 3a). Unexpectedly, this protein showed no significant sequence similarity with known proteins. We found two other similar proteins (Luc2 and Luc3) in our RNA-seq database, although only Luc1 gene was highly expressed in whole body (Fig. 3b). The possible secreted proteins contained 10 cysteine residues, all of them conserved among three proteins, and their molecular weight was estimated to be approximately 35.9 kDa. Because Luc2 and Luc3 were scarcely expressed, we excluded these two genes from further analysis.

### Recombinant GoLuc production and its light emission catalytic activity

**Figure 4:**
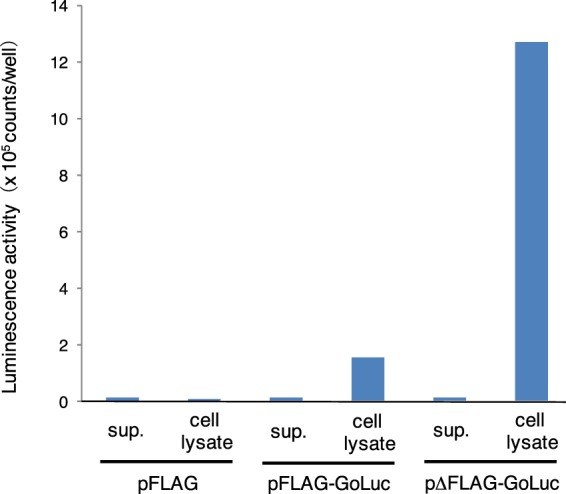
Luminescence activity using recombinant Luc1 protein produced by the mammalian culture cells. Typical luminescence activity data for the cell culture supernatant (sup.) and cell lysate are presented.

**Figure 5:**
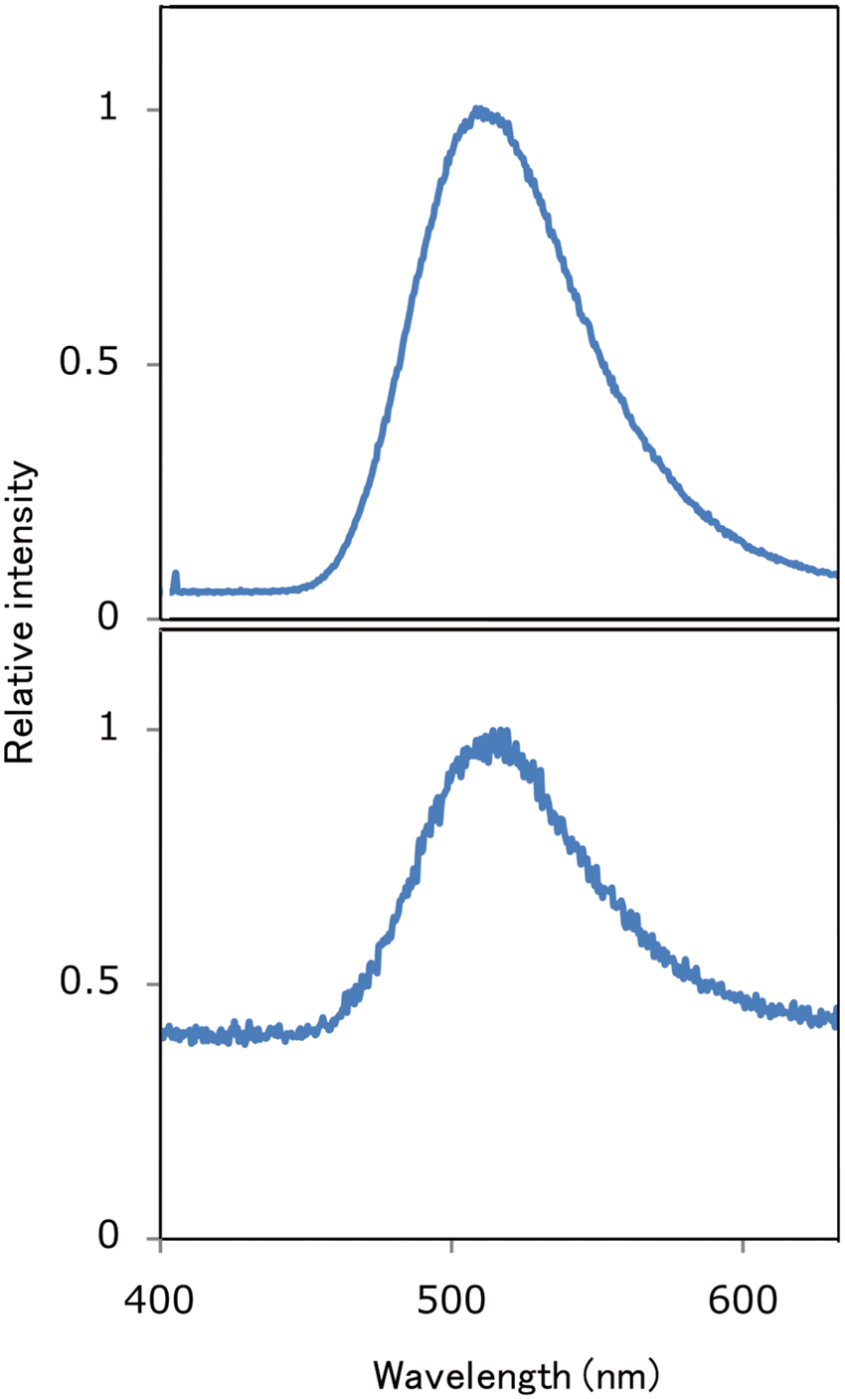
Spectrum analysis of the crude extracts of the fireworm *Odontosyllis undecimdonta* (lower panel) and a mixture of recombinant Luc1 protein and crude luciferin extract (upper panel). The spectral pattern for the latter is noisy because the latter one exhibited lower activity than the former.

To elucidate whether Luc1 is a genuine luciferase or not, we conducted functional analysis by using exogenous expression systems of *E. coli*, mammalian culture cell, and plant cell. Although we could not produce the protein in *E. coli* expression system, we succeeded in producing recombinant Luc1 protein fused with or without FLAG tag by using mammalian COS1 cells. Luminescence activity was observed in the cell lysate fraction, suggesting that the recombinant protein remained inside the cells (Fig. 4). The luminescence activity was much higher when FLAG tag was depleted (Fig. 4, light). Importantly, the emission peak of recombinant Luc1 protein was almost the same as the native luciferase crude extract with a maximum at 510 nm (Fig. 5).

We also expressed codon-optimized form of Luc1 protein fused with avi-tag in plant *Arabidopsis thaliana* and confirmed that the leaf extract expressing the recombinant protein exhibited luminescent activity (Fig. S5).

## Discussion

**Figure 6:**
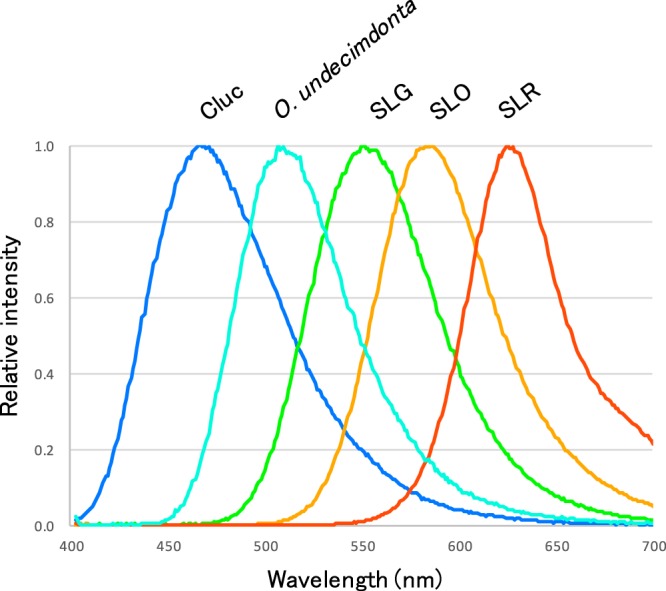
Emission peak of fireworm luciferase in this study compared to luciferases of other animals. Typical emission spectra of commercially available luciferases. Cypridina luciferase system emits blue right (Cluc). Coleoptera and its derivatives emit from green to red color (SLG, SLO, SLR)^18^.

Based on light emission pattern of recombinant protein, Luc1 can be considered to be a genuine fireworm luciferase. Emission peak (~510 nm) of fireworm luciferase is unique compared to those of other animals (Fig. 6)^18^. It is notable that fireworm Luc1 had no sequence homology to any known sequence in public databases. At present, sequence information of annelids, including *Odontosyllis* spp., are scarce. It will be necessary to analyze other species whether they have a similar luciferase gene or not. We could not detect the cross-reactivity using the available luciferase and luciferin as reported previously^15^, suggesting that *O. undecimdonta* had a novel light emission system, including a novel luciferin. Identification of fireworm luciferin deserves future studies.

Recently, the bioluminescence from *O. phosphorea* mucus was suggested to involve a photoprotein rather than a luciferin-luciferase reaction^19^. The bioluminescence reaction among *Odontosyllis* spp. should be examined using recombinant protein from the Japanese species by a cross-reactivity test. Some other marine worms might have a different luminescence system from that of *Odontosyllis* spp. In marine worm belonging to the genus *Chaetopterus*, one of the proteins in the mucus was identified as a member of the ferritin family^20^. Recently, a fluorescent yellow-orange pigment was found in the luminous exudate of another genus, *Tomopteris*^21^. These luminescence systems were suggested to involve a photoprotein.

Many researchers have tried to identify novel luciferase genes from various animals because luciferases with novel properties could lead to new applications. Our findings may extend the utility of luciferin/luciferase system to previously unavailable wavelength range of light.

During the review process of this paper, similar but not identical luciferases from *O. undecimdonta* were reported^22^. The overall strategy using the combination of MS analysis and RNA-seq analysis is almost same as ours’, however, they used conventional protein purification method to obtain luciferase. Most important point of our strategy is the finding of the purity of the luminous mucus. This fact made us be able to identify the luciferase using only a small number of animals.

## Methods

### Animal collection

Marine worms were collected in Toyama Bay on Oct. 2–3, 2013, Oct. 5, 2014, Oct. 4–5, 2015, Oct. 1–2, 2016, and Oct. 7–9, 2017. The moon phases for these days were 27–28, 12, 21–22, 0–1, and 16–17, respectively. The worm length ranged from 5 to 30 mm. The numbers of collected worms were different each year, and ~2,500 individuals were collected over the last 5 years. Animals were maintained in sea water for one to two nights in plastic buckets and were quickly frozen using dry ice for a long-term storage.

### LL reaction of GoLuc

Syllids were crushed using a pipet tip in a 1.5 mL tube containing 200 μL of 50 mM phosphate buffer (pH 8.0). The solution was centrifuged at 20,000 × g for 10 min at 4 °C and was stored at 4 °C until the complete loss of residual activity. Fifty worms were put into 1.5 mL of 99.5% ethanol, and the supernatant after short centrifugation was used as the crude luciferin solution. These solutions were used for activity assays, and the activity was monitored using luminometers, CLX-101 (Toyobo) or Phelios AB-2350 (ATTO). Emission spectrum was measured using a high sensitivity CCD spectrophotometer, AB-1850S (ATTO).

### RNA-seq

The frozen marine worms were subjected to RNA isolation using a MicroPoly(A) purist kit (Thermo Fisher Scientific). RNA-seq analysis was performed as described previously^23^. Using 1 µg of total RNA per sample as the template, cDNA libraries were constructed using the NEBNext mRNA library master mix set for Illumina (Illumina), and the libraries were sequenced by MiSeq (Illumina). The raw reads were subjected to de novo assembly using the Trinity program^24^ implemented in the MASER pipeline (cell-innovation.nig.ac.jp/ public/contents/service.html#pf_maser). After automatic assembly, we manually checked and corrected the target gene sequences using the Integrative Genomics Viewer^25^. Sequence read mapping was performed using the BWA-mem software^26^ implemented in the MASER pipeline, whereby the transcript expression levels were estimated to calculate the fragments per kilobase of exon per million (FPKM) values.

### De novo amino acid sequencing

The luminous mucus was collected from five individual animals and subjected to SDS-PAGE. The band stained with Coomassie Brilliant Blue was cut from the gel and destained with 50% acetonitrile. The gel was then incubated at 56 °C for 30 min and then an incubation at room temperature for 45 min in 50 mM iodoacetamide and 100 mM NH_4_HCO_3_. The gel was washed with 100 mM NH_4_HCO_3_, dried in a vacuum chamber, and subjected to trypsin digestion at 37 °C for 16 h. The peptide fragments after trypsin digestion were desalted with ZipTip C18 (Millipore) and subjected to MS analysis (ultra TOF/TOF, Bruker Daltonics).

### GoLuc cloning

In parallel with the RNA-Seq analysis, we constructed a cDNA library using the Smart cDNA library construction kit (Clontech). *GoLuc* was amplified using ExTaq with the primers od-13 (5′-CATATGAAGTTAGCACTGTTACTCAGC-3′) and od-14 (5′-TCTAGACTGTTGTAGGTTATACATCTCAGC-3′). The PCR cycles were repeated for 30 times with the following temperatures: 98 °C for 10 s, 55 °C for 30 s, and 72 °C for 1 min. The PCR product was inserted into the pCR4.0-Topo vector (Thermo Fisher Scientific) to produce pCR-GoLuc. The sequence of pCR-GoLuc was analyzed using the 3130xl genetic analyzer (Applied Biosystems) and was deposited as LC333365 in the DDBJ/EMBL/GenBank data bases (Fig. S6). pCR-GoLuc was used as a template for the PCR using KOD plus neo (Toyobo) to produce the mammalian expression vector with the primers od-17 (5′- TCTAGACTGTTGTAGGTTATACATCTCAGC -3′) and od-19 (5′- AACCCGGGTTACTGTTGTAGGTTATACAT -3′). The PCR product was digested with HindIII and SmaI and ligated to the same restriction enzyme sites of pFLAG-CMV-2 (Sigma Aldrich) to produce pFLAG-GoLuc. This plasmid was used as an inverse PCR template and amplification was performed using KOD plus neo with the primers od-22 (5′-ATGAAGTTAGCACTGTTACTC-3′) and od-23 (5′-GGTAGATCAATTCTGACGGTT-3′). The PCR product was self-ligated to obtain pΔFLAG-GoLuc. This plasmid was designed to express recombinant GoLuc without the FLAG tag.

### Sequencing of the genome region encoding GoLuc

*O. undecimdonta* genome DNA was obtained using the DNeasy plant mini kit (Qiagen) according to the manufacturer’s manual. PCR amplification was performed using the genomic DNA as a template and ExTaq with the primers od-13 and od-14. PCR cycles of 98 °C for 10 s, 55 °C for 30 s, and 72 °C for 6 min were repeated 30 times. The PCR product was cloned into the pCR4.0-Topo vector, and the resulting plasmid was sequenced. The obtained sequence was deposited as LC333366 in the DDBJ/EMBL/GenBank data bases (Fig. S7).

### GoLuc expression in mammalian cells

COS1 cells were cultured in Dulbecco’s modified Eagle’s medium (DMEM, Wako) supplemented with 10% fetal bovine serum (FBS) at 37 °C in a humidified incubator exposed to 5% CO_2_. COS1 cells were transfected with each expression plasmids using Lipofectamine 3000 (Invitrogen). On the following day, the culture medium was replaced with serum-free DMEM. The culture medium was collected 24 h after medium exchange and used as the supernatant fraction. Cells were lysed with 10 mM Tris-HCl (pH 7.4) containing 0.05% NP40 using sonication. The lysate was centrifuged at 20,000 g for 5 min, and the resulting supernatant was used as the cell extract fraction. The activity of recombinant GoLuc in each fraction was measured by adding NaCl and MgCl_2_ solutions to be a final concentration 300 mM and 20 mM each, and luciferin solution. The luminescence of the mixture was immediately measured using a Phelios AB-2350 luminometer (ATTO). We filed an international patent of this protein features as WO/2017/155036 at 9 Mar., 2017, and this patent was published at 14 Sep., 2017^27^.

### GoLuc expression in plant

GoLuc gene was codon-optimized for plant and chemically synthesized (plant GoLuc). The plant GoLuc fragment was inserted into the vector for expressing fusion protein with avi-tag in plant (p35SaviHSPG) and the resultant construct harboring promoter, gene, and terminator was transferred to pBCKK T-DNA vector^28^ by Gateway LR reaction. P35SaviHSPG vector was prepared by inserting chemically synthesized avi-tag fragment into p35SHSPG vector^29^. *Arabidopsis thaliana* Columbia-0 (Col-0) strain was transformed by floral-dip method^30^. T1 transgenic lines were selected on the media containing kanamycin and single true leaf taken from 3-wk old plants was homogenized in 200 μL lysis buffer provided by Promega Inc. and 10 μL aliquot was added into luciferin solution to measure luminescence.

## Electronic supplementary material

Dataset 1
