# Supplementary material for: Novel gene encoding a unique luciferase from the fireworm Odontsyllis undecimdonta
Source: Sci Rep. 2018 Aug 24;8:12789. doi: 10.1038/s41598-018-31086-1 (PMC6109096; doi:10.1038/s41598-018-31086-1)
Supplement: Supplementary file 1 — Dataset 1 [file 41598_2018_31086_MOESM1_ESM.pdf]

# Novel gene encoding a unique luciferase from the fireworm *Odontsyllis undecimdonta*

Yasuo Mitani, [1] Rie Yasuno, [2] Minato Isaka, [1,3] Nobutaka Mitsuda, [1,3] Ryo Futahashi, [1] Yoichi Kamagata, [1] and Yoshihiro Ohmiya [2,4]*

[1]Bioproduction Research Institute, National Institute of Advanced Industrial Science and Technology (AIST), Tsukuba 305-8566, Japan; [2]Biomedical Research Institute, AIST, Tsukuba 305-8566, Japan; [3]Graduate School of Science and Technology, Saitama University, Saitama 338-8570, Japan; [4]DAILAB, Biomedical Research Institute, AIST, Tsukuba 305-8566, Japan

**Supplementary information**

Fig. S1. Mass spectrum analysis of the GoLuc protein extracted from a gel. The gel fragment that exhibited luminescence activity was treated with trypsin and subjected to MS analysis. Two major peaks at m/z = 970.5 and 1075.5 were selected for the following MS/MS analysis.

Fig. S2. MS/MS analysis of the peak at 970.5 in the MS analysis. MS/MS data for the 970.5 peak were analyzed and a peptide sequence, NVVPLWSR, was deduced from the mass differences.

Fig. S3. MS/MS analysis of the peak at 1075.5 in the MS analysis. MS/MS data for the 1075.5 peak were analyzed, and the peptide sequences, WEDWVNAR and WEDDAVNAR, were deduced from the mass differences.

Fig. S4. Schematic illustration of the genome region encoding GoLuc. GoLuc is encoded by 8 exons. Nucleotide positions and amino acid positions corresponding to the GoLuc sequence are shown by the numbers (nt.) and (a.a.), respectively.

Fig. S5. Relative luminescence of plant leaf extract containing plant GoLuc. Codon-optimized GoLuc for plant was stably expressed in A. thaliana plants and relative luminescence of leaf extract was measured in the 12 independent transgenic plants. “wt” represents wild type.

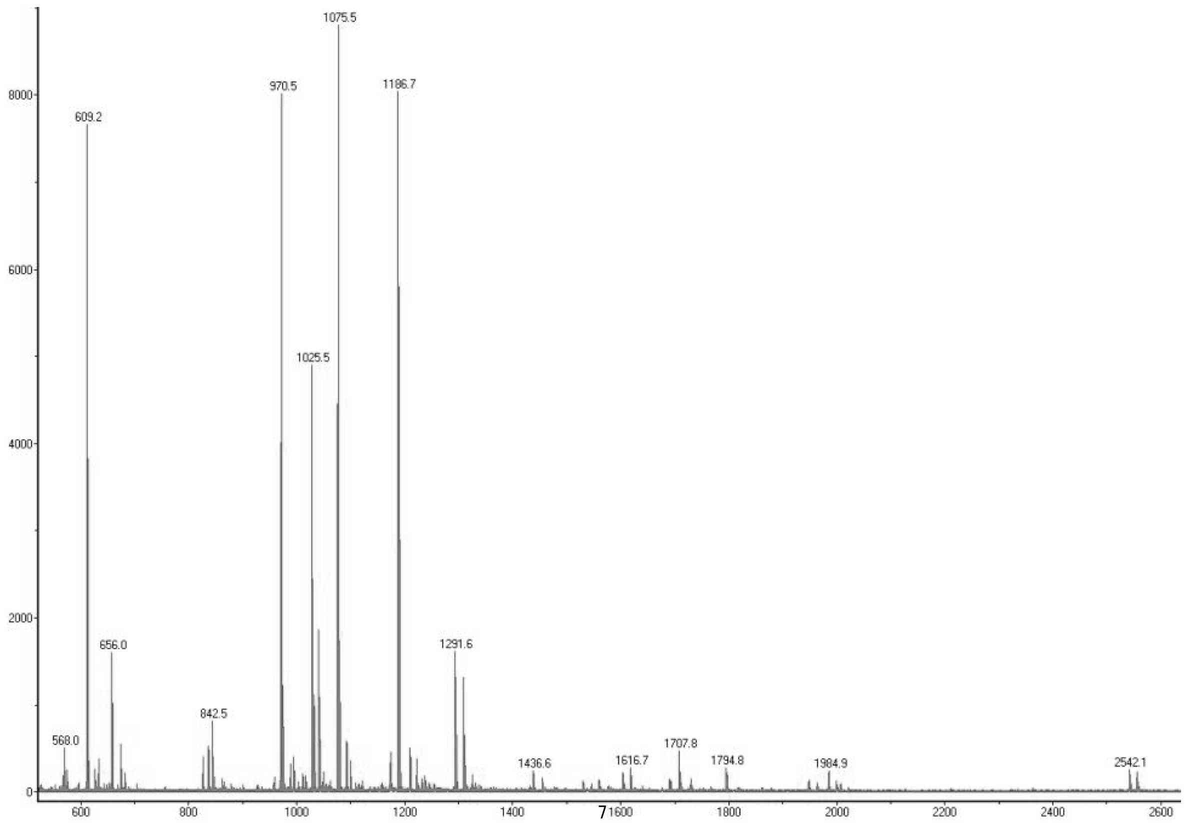


Fig. S1 Mass spectrum analysis of GoLuc protein extacted from a gel.

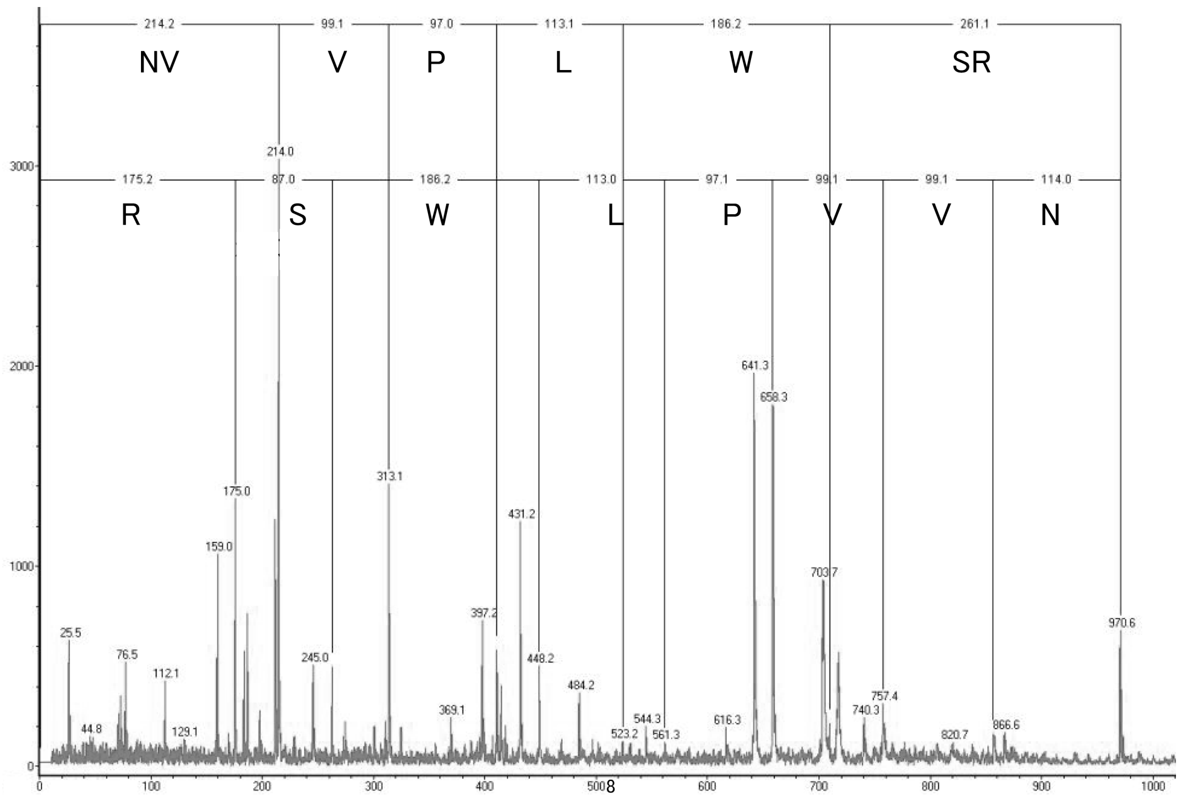


Fig. S2 MS/MS analysis of the peak at 970.5 in the MS analysis.

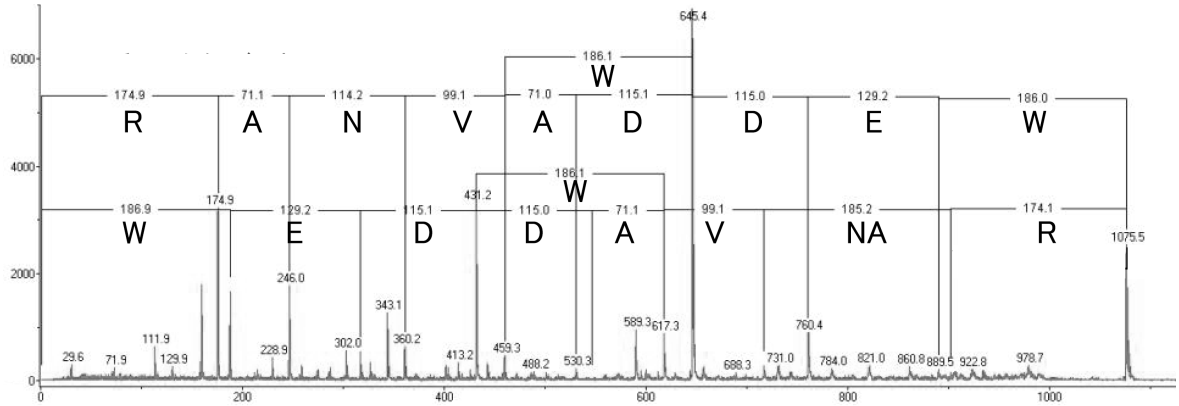


Fig. S3 MS/MS analysis of the peak at 1075.5 in the MS analysis.

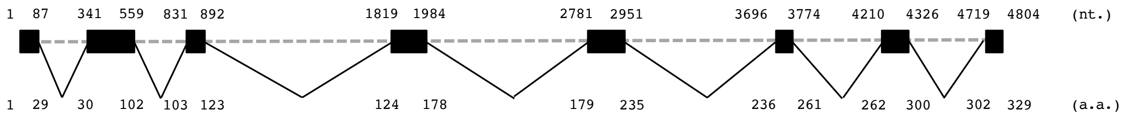


Fig. S4 Shematic illustration of the genome region encoding for GoLuc gene.

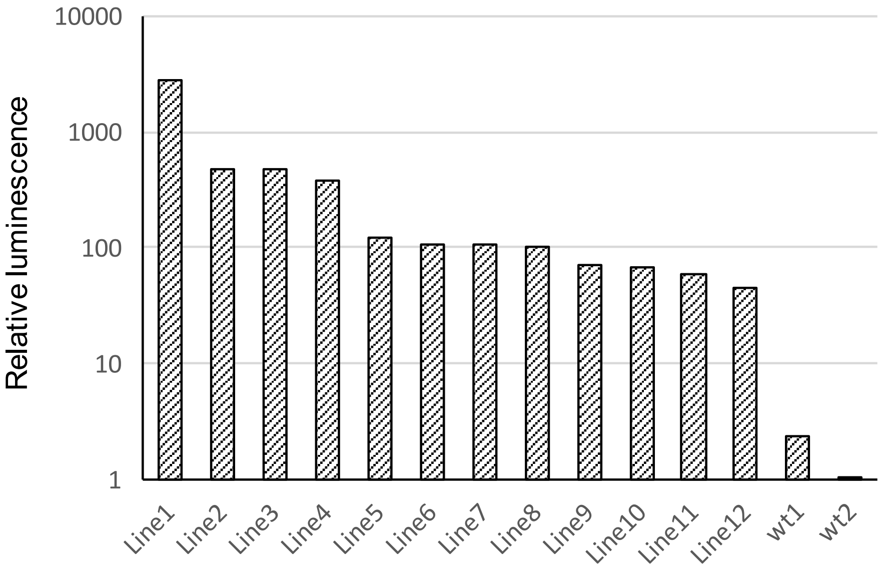


Fig. S5 Relative luminescence of plant leaf extract containing plant GoLuc.
